# Achieving WHO's Goal for Reducing Cesarean Section Rate in a Chinese Hospital

**DOI:** 10.3389/fmed.2021.774487

**Published:** 2021-11-22

**Authors:** Yan-Jie Ji, Hai-Bo Wang, Zhi Bai, Da-Jian Long, Kaidong Ma, Jie Yan, Yun-Xiu Li, Yang-Feng Wu, Huixia Yang

**Affiliations:** ^1^The First Dongguan Affiliated Hospital of Guangdong Medical University, Dongguan, China; ^2^Peking University Clinical Research Institute, Peking University First Hospital, Beijing, China; ^3^Shenzhen Longhua District Central Hospital, Shenzhen, China; ^4^Department of Obstetrics and Gynecology, Peking University First Hospital, Beijing, China

**Keywords:** health policy, policy implementation, China, cesarean section rate, one-child policy

## Abstract

**Background:** To address the worldwide dramatically increased Cesarean section (CS) rate in the past decades, WHO has recommended the CS rate should not be higher than 10–15%. Whether it is achievable remains unknown.

**Methods:** We collected the data of delivery from 2008 to 2017 in two typical regional hospitals in China: Longhua Hospital (national policies rigorously implemented) and Dongguan Hospital (national policies not rigorously implemented). We compared between the two hospitals the 10 years trend in annual rate of CS, standardized by age, education level, parity, and CS history, against the time of issuing relevant national, local, and hospital policies.

**Results:** In 10 years, 42,441 women in Longhua and 36,935 women in Dongguan have given birth. China's first national policy on CS reduction was issued in 2010 and the formal relaxation of one-child policy was issued in 2015–2016. In Longhua, the standardized annual CS rate was around 35% in 2008–2009, which declined sharply since 2010 down to 13.1% in 2016 (*p* < 0.001) and then leveled off. In contrast, in Dongguan, the rate stayed around 25% at the beginning, increased to 36% in 2011, decreased sharply to 27% in 2012, and leveled off until 2015 (*p* < 0.001), and then bounced back to 35% in 2017. The proportion of women with the history of CS increased significantly in the two hospitals (both roughly from 6% before 2010 to 20% after 2015). Analyses stratified by modified Robson classification showed that CS rates reduced in all risk classes of delivery women in Longhua but only in the Robson class 2 group in Dongguan. Major complications did not differ by hospital.

**Conclusion:** With vigorously implementing national policies at micro levels, the WHO-recommended CS rate could be achieved without increase in major complications.

## Introduction

Although Cesarean sections (CSs) with medical indication can effectively reduce maternal and neonatal morbidity and mortality, overuse of CS is a threat to the health of the mother and the child (1, 2). WHO has recommended that the reasonable CS rate should not be higher than 10–15% of all deliveries (3, 4). In contrary to the recommendation, the worldwide CS rate has increased dramatically during the past 30 years and has led to global concerns (5, 6). This significant increase has been driven mainly by non-medically indicated CS in many countries (7).

In China, the overall annual CS rate increased from 29% in 2008 to 35% in 2014 (8) as one of the highest CS rates in the world (9). Concerning about the “alarming” CS rates, the Chinese government issued a variety of national policies, programs, and activities to reduce the CS rate nationwide (8). As a result, China became the only country where a reduction in CS rate has been achieved (6, 10, 11). The data from China's National Maternal Near Miss Surveillance System (NMNMSS) showed that hospital-based CS rate decreased from 45% in 2012 to 41% in 2016 (10). While we are proud of the big success as the first country in the world to have CS rate reduced at the country level, we have to admit that the CS rate (over 40% on an average) is still too high and far beyond the ideal rate of 10% as recommended by the WHO (4). Whether the WHO-recommended CS rate can be achieved in China and how it can be achieved remain largely unknown.

In addition to the national policies and activities that have been shown effective to reduce the national CS rate in China (8, 10, 12), the local government and hospital policies are also critical to make the national policies implemented and sustained. However, evidences on the relationship between the CS rate and the implementation of the national policies at hospital levels are lacking. Understanding this relationship would provide insights for the development of targeted intervention measures that will further bring down the high national CS rate in China.

China's one-child policy was first relaxed in November 2013 (10, 13) and then the two-child policy was implemented in October 2015 (13). How the policy relaxation would impact on the trend in overall CS rate in China bears critical importance but remains to be understood. On the one hand, the policy relaxation would accelerate the declining trend of CS rate among nulliparous women for they might not intend to have CS, so that they keep at lower risk for future births. On the other hand, multiparous women with higher age and previous uterine scar would have increased because of the previous one-child policy and high CS rate, they might be intended to choose CS for the birth (14).

Therefore, the current study aimed to understand, under the national CS reduction movement, the discrepant influences of the local policies and actions at hospital level on the CS rate, by comparing two typical regional hospitals in China for 10 years with against to the time of issuing national policies/clinical guidelines. Also, we explored how the relaxation of the one-child policy would impact the trend of CS rate in subgroups by parity and CS history in each hospital. The selected two hospitals (Shenzhen Longhua District Central Hospital, Longhua Hospital; The First Dongguan Affiliated Hospital of Guangdong Medical University, Dongguan Hospital) are located adjacent to each other but administratively belonged to different cities of Guangdong Province, which had significant difference in responses to the national policies and clinical guidelines on the reduction of CS rate. It would be helpful to analyze the effect of policy implementation on CS rate assuming the similar population characteristics in these two hospitals.

## Materials and Methods

### Setting and Study Population

This was a retrospective cohort study conducted in two hospitals in Guangdong Province of China: Longhua Hospital located in Shenzhen and Dongguan Hospital located in Dongguan, which is next to Shenzhen. The two hospitals are both tertiary hospitals and about 50 kms apart, though administratively belong to different cities. Shenzhen is one of the China's four first-tier cities, with an estimated population of 21 million, birth rate of 25‰, 2.7 medical staffs per thousand population, and gross domestic product per capita of $28,500. Dongguan is a third-tier city in China, with an estimated population of 8 million, birth rate of 22‰, 2.1 medical staffs per thousand population, and gross domestic product per capita of $14,100 (15).

We retrieved data from the hospital information systems for the obstetric records from January 1, 2008 to December 31, 2017. The study participants included women who aged ≥18 years, delivered at a gestational age of ≥28 weeks, and gave birth with a fetus of birth weight 1,000 g or higher. The study was approved by the institutional review boards of the two participating hospitals (IRB2020-135-01). Informed consent was not obtained as this was a retrospective study and the data came from the hospital's medical records.

### Data Collection and Study Outcomes

The downloaded data were reviewed to ensure that the inclusion and exclusion criteria were met. Then, the database was de-identified and used for further analyses. Data abstracted included maternal age, gestational age, parity, history of CS, fetal position, delivery mode, and outcomes of delivery including neonatal death and maternal complications.

The primary outcome was the annual CS rate standardized by age, education level, parity, and history of CS. Secondary outcomes included CS rate in subgroups separated by parity, history of CS, and Robson classification.

We modified the Robson classification into the following eight groups, as information on induction of labor was not available in the study: (1) nulliparous, single cephalic pregnancy, ≥37 weeks' gestation; (2) multiparous, single cephalic pregnancy, ≥37 weeks' gestation without a uterine scar; (3) multiparous, single cephalic pregnancy, ≥37 weeks' gestation with uterine scar(s); (4) nulliparous, single breech pregnancy; (5) multiparous, single breech pregnancy; (6) all multiple pregnancies, with/without previous uterine scar(s); (7) single pregnancy in other abnormal lie, with/without previous uterine scar(s); and (8) single cephalic pregnancy, ≤ 36 weeks' gestation (10, 16). We further merged the Robson groups 4 to 7 into one group, since these groups accounted for only 4.0% of all obstetric women and 13.9% of all CS cases.

Maternal complications were classified into mutually exclusive categories, including hysterorrhexis, hysterectomy, blood transfusion, and maternal deaths. Maternal death was defined as death from any cause during pregnancy or within 42 days of termination of pregnancy, except for accidental deaths. Neonatal death was defined as any neonatal deaths in the first 6 days after birth among stillbirths.

### Statistical Analysis

We used Statistics Analysis System software (version 9.3, SAS Institute, Cary, NC, USA) for statistical analysis. All study variables were categorical and the differences between the two study hospitals were compared using the χ^2^ test. Using all women in the two study hospitals as the standard population, we calculated the weighted average annual CS rate standardized by age, education level, parity, CS history, and Robson classification over the 10 years based on the weights from combined population, to compare the trends between the two study hospitals. The CS rates were tested for changes over time using χ^2^ tests for trend. Crude odds ratio (cOR) and 95% CI were calculated for the association between year and the proportion of nulliparous women, with truncation analysis at year 2014. To understand the change in 10 years in composition of women in type of delivery by parity and CS history, we plotted the data annually. Similarly, we plotted the annual composition of CS cases by parity and CS history to understand the 10 years change, which indicated the change of each group's contribution to the overall CS rate. A *p* < 0.05 was considered to be statistically significant.

### Role of the Funding Source

The funder of the study had no role in study design, data collection, statistical analysis, results interpretation, or writing of the manuscript. All authors had full access to all the data in the study and accept final responsibility to submit for publication.

## Results

### Differences in the Two Hospitals in Responses to the National Policies and Clinical Guidelines on Reduction of CS Rate

Against to each national policy and guideline, Table 1 listed the local policies and actions taken for the implementation. It clearly indicated that local health authorities and the director of obstetrics and gynecology department performed very differently in the two study hospitals. In Longhua, specific local policies, actions, and department rules were developed and taken to reinforce the national policies and guidelines recommendations. In Dongguan hospital, no specific local policies or department rules were taken except a research program that was initiated since 2012 to reduce CS rate among multiparous women with a scar uterus.

**Table 1:** The relevant national policies/clinical guidelines and local policies and actions for the implementation.

**Year**	**Key provisions in national policies and clinical guidelines**	**Issuing body**	**Local policies and actions for the implementation**	
			**Longhua hospital**	**Dongguan hospital**
2010	• WHO report: China's CS rate was one of the highest in the world (9)	WHO	• Start to train the team of and promote the use of midwifery skills	• Responded to the nation's call to reduce CS through group meetings and communications, but no specific actions taken.
	• National programme to promote vaginal delivery and improve maternal and infant health (Jun, 2010) ✓ Establish training centers to promote midwifery techniques for improvement of maternal and infant health ✓ Selection and appraisal of hospitals and departments with excellence in promoting virginal delivery	CMCHA		
	• National protocol for performance assessment of maternal and child health services at county level (Dec, 2010) ✓ Reduce CS rate without medical indication ✓ Non-medical CS is listed as an indicator for performance appraisal of hospitals	MOH		
2011	• Guidelines of maternal health management services (Apr, 2011) ✓ Encouraging vaginal delivery ✓ Reducing CS without medical indications	MOH		• Initiate research on the method to reduce CS among multiparous women with a scar uterus.
2012	• Implementation plan of the Chinese women and children development guidelines in 2011–2020 (Feb, 2012) ✓ Promote spontaneous labor ✓ Encourage vaginal delivery ✓ Strengthen quality of midwifery skills	MOH	• Local health authority monitor the non-medical CS rate, on quarterly basis. • Non-medical CS should be approved by the department director.	• Local health authority listed the non-medical CS rate as an indicator of hospital performance, but no site-monitoring.
2013	• Partial two child policy was issued (Dec, 2013)	SC		
2014	• Notice on carrying out the review of the baby friendly hospital (Aug, 2014) ✓ List the number of non-medical CS as an indicator to assess the hospital performance ✓ Non-medical CS should be reviewed by hospital director	NHFPC (former MOH)	• Strict implementation, trial of vaginal birth was required for women with <2 times of CS	
	• The expert consensus on cesarean delivery operation (Nov, 2014) ✓ Updated medical indications for CS ✓ Protect doctor's right to refuse non-medical CS	CSOG		
2015	• Fully two child policy officially released (Oct, 2015)	SC		
2016	• The expert consensus on the management of vaginal birth after CS (VBAC) (Aug, 2016) ✓ Indications and contradictions of VBAC was issued ✓ Clinical guidelines on implementing VBAC was issued.	CSOG		• In early 2017, a case with uterus rapture took place and went for law suit.

*WHO, World Health Organization; CMCHA, China Maternal and Child Health Association; MOH, Ministry of Health; NHFPC, National Health and Family Planning Commission; CSOG, Chinese Society of Obstetrics and Gynecology; SC, State Council*.

### Comparison of the Two Study Hospitals in Characteristics of Delivery Women

From 2008 to 2017, a total of 79,376 women gave birth in the two study hospitals; 42,441 in Longhua and 36,935 in Dongguan. The proportion of women aged over 35 years and the proportion of women in the modified Robson classification group 3–8 were comparable between the two hospitals. However, women in Longhua were more educated and likely to be nulliparous, and less likely to have CS, in comparison with women in Dongguan (Table 2).

**Table 2:** Demographical and clinical characteristics of women delivering babies in 2008–2017 in two hospitals in Guangdong, China.

**Characteristics**		**Longhua (*N* = 42,441)**	**Dongguan (*N* = 36,935)**	***P*-value**
Year of delivery	2008–09	6,497 (15.3)	8,395 (22.7)	<0.0001
	2010–11	7,782 (18.3)	8,403 (22.7)	
	2012–13	9,229 (21.8)	7,962 (21.6)	
	2014–15	8,913 (21.0)	6,141 (16.6)	
	2016–17	12,020 (23.6)	6,034 (16.3)	
Age (Years)	<35	38,915 (91.7)	33,926 (91.9)	0.41
	≥35	3,526 (8.3)	3,009 (8.1)	
Education (Years)	<9	14,310 (33.7)	23,797 (64.4)	<0.0001
	≥9	28,131 (66.3)	13,138 (35.6)	
Gestational age (Weeks)	28–33	472 (1.1)	521 (1.4)	<0.0001
	34–36	1,783 (4.2)	1,348 (3.7)	
	≥37	40,186 (94.7)	35,066 (94.9)	
Parity	Nulliparous	19,763 (46.6)	15,171 (41.1)	<0.0001
	Multiparous	22,678 (53.4)	21,764 (58.9)	
	Without uterine scar	17,000 (75.0)	17,214 (79.1)	<0.0001
	Virginal delivery	15,480 (91.1)	14,931 (86.7)	<0.0001
	Cesarean birth	1,520 (8.9)	2,283 (13.3)	
	With uterine scar	5,678 (25.0)	4,550 (20.9)	
	Virginal delivery	1,639 (28.9)	832 (18.3)	<0.0001
	Cesarean birth	4,039 (71.1)	3,718 (81.7)	
Modified Robson classification	Nulliparous, single, cephalic, ≥37 weeks	18,027 (42.5)	13,790 (37.3)	<0.0001
	Multiparous, single, cephalic, ≥37 weeks, without uterine scar	15,755 (37.1)	15,876 (43.0)	
	Uterine scar, single, cephalic, ≥37 weeks	5,166 (12.2)	4,130 (11.2)	
	Nulliparous, single, breech	579 (1.4)	551 (1.5)	
	Multiparous, single, breech, including those with uterine scar	513 (1.2)	597 (1.6)	
	All multiple pregnancies, including those with uterine scar	335 (0.8)	367 (1.0)	
	All single, other abnormal lies, including hose with uterine scar	78 (0.2)	132 (0.4)	
	All single, cephalic, ≤ 36 weeks, including those with uterine scar	1,988 (4.7)	1,492 (4.0)	

### Trend in Composition of Delivery by Parity and History of CS

**Figure 1:**
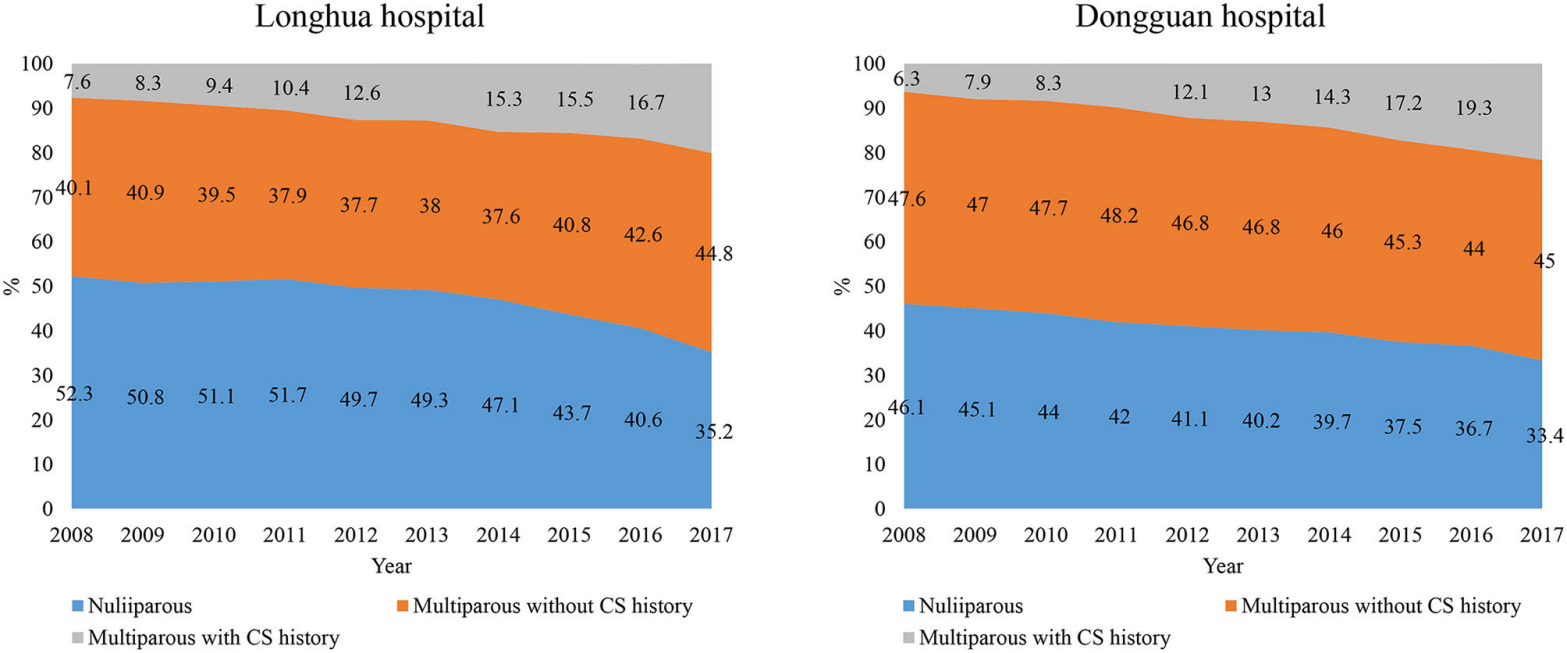
Trends in composition of women by parity and CS history in 10 years from 2008 to 2017 in two hospitals.

Although the number of deliveries increased in Longhua but decreased in Dongguan during the 10 years, the composition by parity were similar and showed the same temporal trend in the two hospitals. The proportion of nulliparous women decreased slowly from 2008 to 2014 (from 52.3 to 47.1% in Longhua, cOR 0.97, 95% CI: 0.96–0.98; and from 46.1 to 39.7% in Dongguan, cOR 0.95, 95% CI: 0.94—.97) but more quickly from 2015 to 2017 (from 43.7 to 35.2% in Longhua, cOR 0.84, 95% CI: 0.80–0.87; and from 37.5 to 33.4% in Dongguan, cOR 0.91, 95% CI: 0.87–0.96) (Figure 1). Among multiparous women, the proportion with CS history in both the hospitals increased with a similar magnitude, from 7.6 to 20.1% in Longhua and from 6.3 to 21.7% in Dongguan (Figure 1).

### Trends in CS Rates

**Figure 2:**
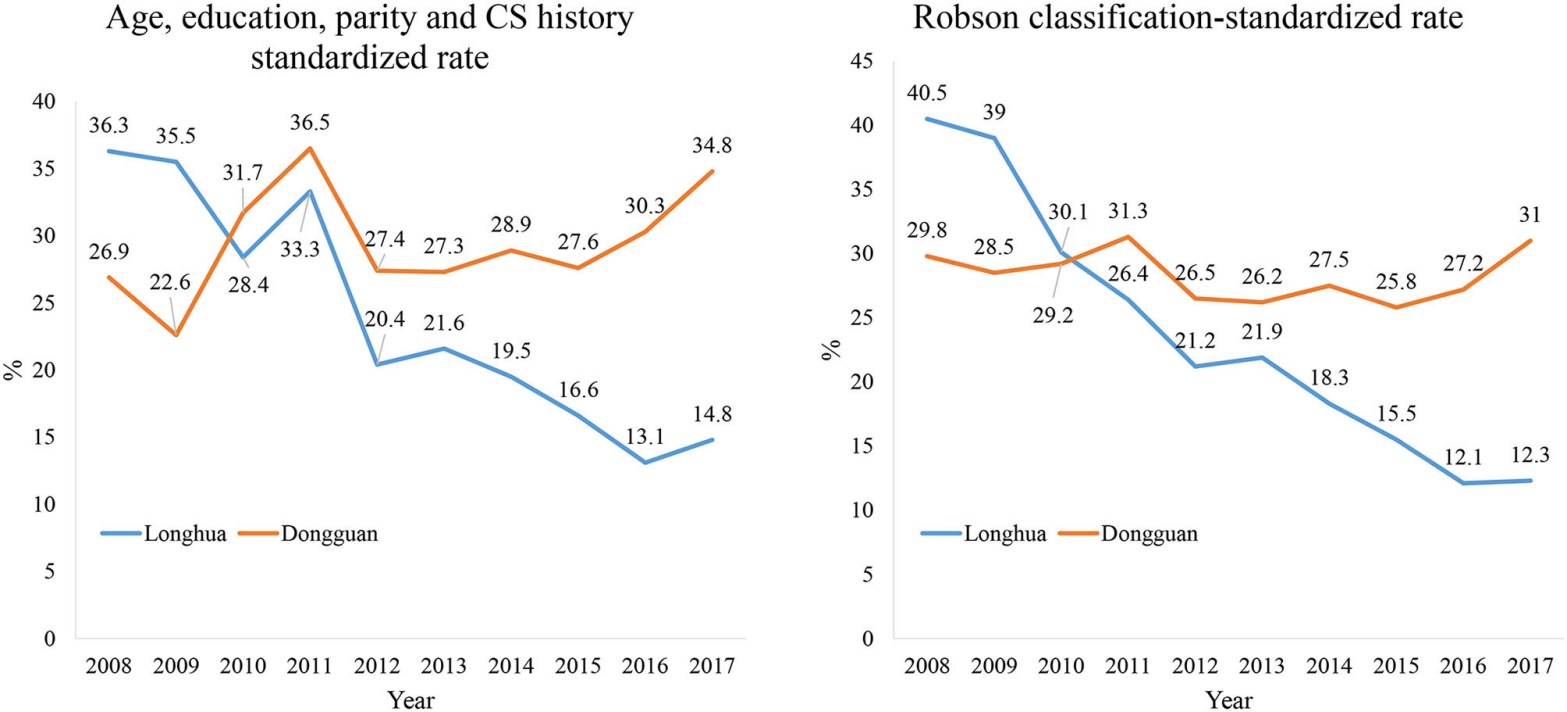
Comparison of two hospitals in CS rate standardized for composition of age, education level, parity, and history of CS (Left) and Robson classification (Right).

Overall, 24.8% of women in the two hospitals had CS. The standardized CS rates in Longhua stayed around 35% at the beginning and started to decrease steadily and sharply from the year 2010 until the year 2016 (13.1%) and then leveled off (*p* for trend < 0.001). The standardized CS rate in Dongguan varied around 25% at the beginning, increased to 36% in 2011, decreased sharply to 27% in 2012 and leveled off until 2015 (*p* < 0.001), and then bounced back to 35% in 2017 (Figure 2). In fact, the CS rate declined significantly over the 10 years period in all subgroups by parity and history of CS in Longhua, while in Dongguan, the CS rate only declined from 2011 to 2015 and only in multiparous women (Table 3).

**Table 3:** CS rates (%) in each year in all women and by parity and history of CS in two study hospitals.

											**Multiparous**									
**Year**	**All women (*****N*** **=** **79,376)**					**Nulliparous (*****N*** **=** **34,934)**					**Without CS history (*****N*** **=** **34,214)**					**With CS history (*****N*** **=** **10,228)**				
	**Longhua**		**Dongguan**			**Longhua**		**Dongguan**			**Longhua**		**Dongguan**			**Longhua**		**Dongguan**		
	**(*****N*** **=** **42,441)**		**(*****N*** **=** **36,935)**			**(*****N*** **=** **19,763)**		**(*****N*** **=** **15,171)**			**(*****N*** **=** **17,000)**		**(*****N*** **=** **17,214)**			**(*****N*** **=** **5,678)**		**(*****N*** **=** **4,550)**		
	***n***	**%**	***n***	**%**	***P***	***n***	**%**	***n***	**%**	***P***	***n***	**%**	***n***	**%**	***P***	***n***	**%**	***n***	**%**	***P***
2008	1,260	37.9	1,156	25.8	<0.001	688	39.5	545	26.4	<0.001	324	24.3	355	16.7	<0.001	248	98.0	256	91.1	<0.001
2009	1,182	37.3	979	25.0	<0.001	625	38.8	440	24.9	<0.001	297	22.9	252	13.7	<0.001	260	99.2	287	92.9	<0.001
2010	1,044	28.1	1,100	26.2	0.05	501	26.4	482	26.1	0.81	200	13.6	283	14.1	0.69	343	98.6	335	95.4	0.02
2011	1,041	25.6	1,206	28.7	0.001	466	22.1	521	29.6	<0.001	175	11.3	296	14.6	0.004	400	94.8	389	94.0	0.60
2012	1,040	21.4	1,164	26.2	<0.001	409	16.9	511	28.0	<0.001	132	7.2	271	13.0	<0.001	499	81.4	382	71.1	<0.001
2013	950	21.8	924	26.2	<0.001	389	18.1	361	25.5	<0.001	119	7.2	217	13.2	<0.001	442	79.4	346	75.5	0.15
2014	936	20.1	908	27.7	<0.001	344	15.7	373	28.7	<0.001	91	5.2	170	11.3	<0.001	501	70.3	365	77.8	0.004
2015	689	16.2	779	27.2	<0.001	216	11.6	297	27.6	<0.001	60	3.4	126	9.7	<0.001	413	62.6	356	72.2	<0.001
2016	599	12.4	924	30.7	<0.001	164	8.4	337	30.5	<0.001	56	2.7	140	10.6	<0.001	379	47.0	447	76.8	<0.001
2017	758	14.6	1,067	35.3	<0.001	138	7.5	339	33.6	<0.001	66	2.8	173	12.7	<0.001	554	53.1	555	84.6	<0.001
P_trend_	—	<0.001	—	<0.001	—	—	<0.001	—	<0.001	—	—	<0.001	—	<0.001	—	—	<0.001	—	<0.001	—
All	9,499	22.4	10,207	27.6	—	3,940	19.9	4,206	27.7	—	1,520	8.9	2,283	13.3	—	4,039	71.1	3,718	81.7	—

**Figure 3:**
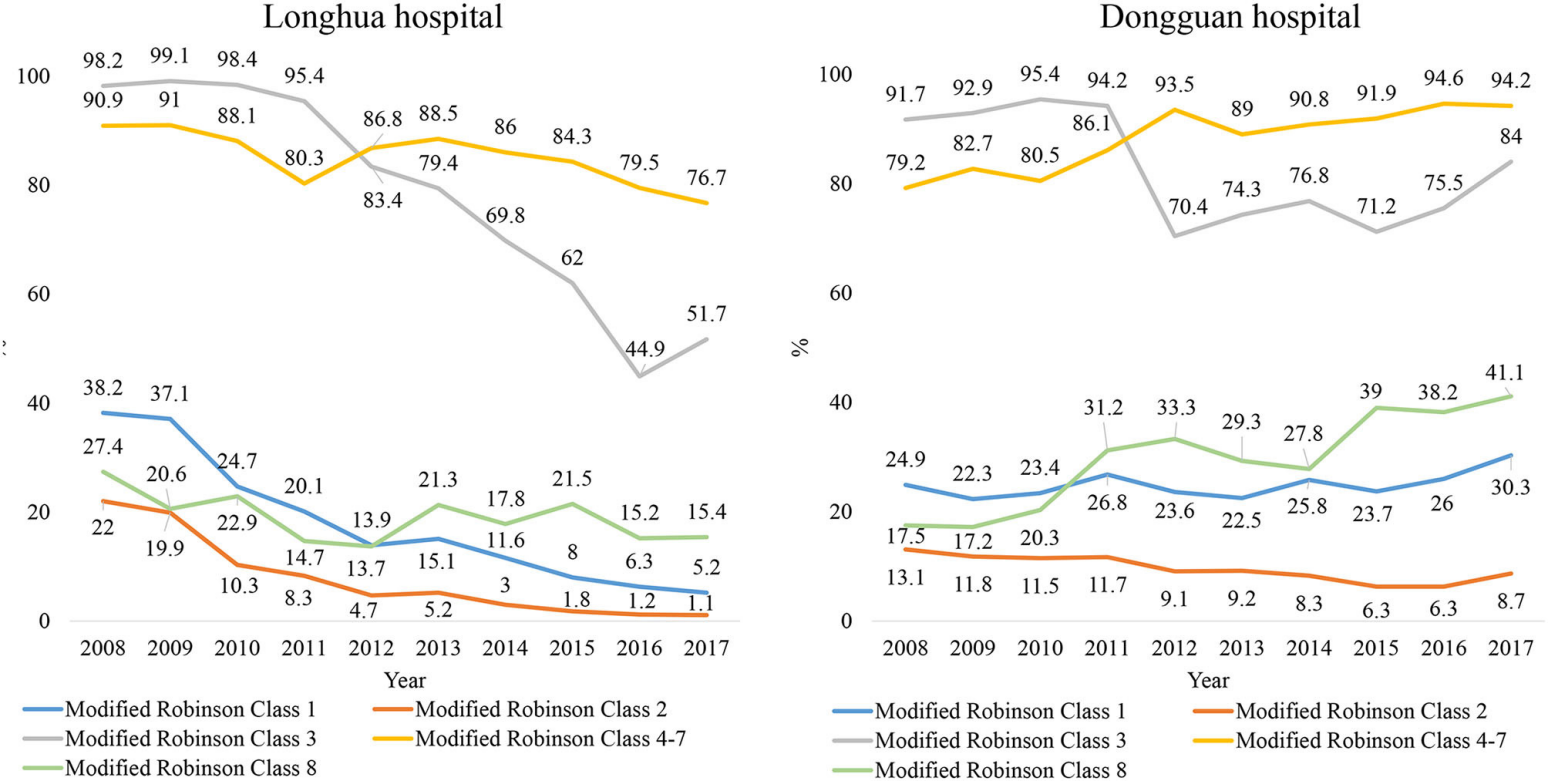
Comparison between Longhua and Dongguan hospitals on trends in CS rate in 10 years from 2008 to 2017, by Robson classification. Robson' class 1: Nulliparous, single, cephalic, ≥37 weeks; Robson's class 2: Multiparous, single, cephalic, ≥37 weeks, without uterine scar; Robson's class 3: Uterine scar, single, cephalic, ≥37 weeks; Robson's class 4–7 (Nulliparous, single, breech/multiparous, single, breech/all multiple pregnancies/all single other abnormal lies; Robson's class 8: All single, cephalic, ≤ 36 weeks.

Figure 3 showed temporal trends of CS rates in each subgroup by modified Robson classification. Again, there was a significant decline of CS rate in all subgroups in Longhua Hospital, particularly in the Robson class 1 group (from 38.2 to 5.2%). However, the CS rate declined only among women in Robson class 2 and 3 groups in Dongguan, and it even increased in Robson class 4 to 8 groups.

### Number of CS Attributed to Each Subgroup

**Figure 4:**
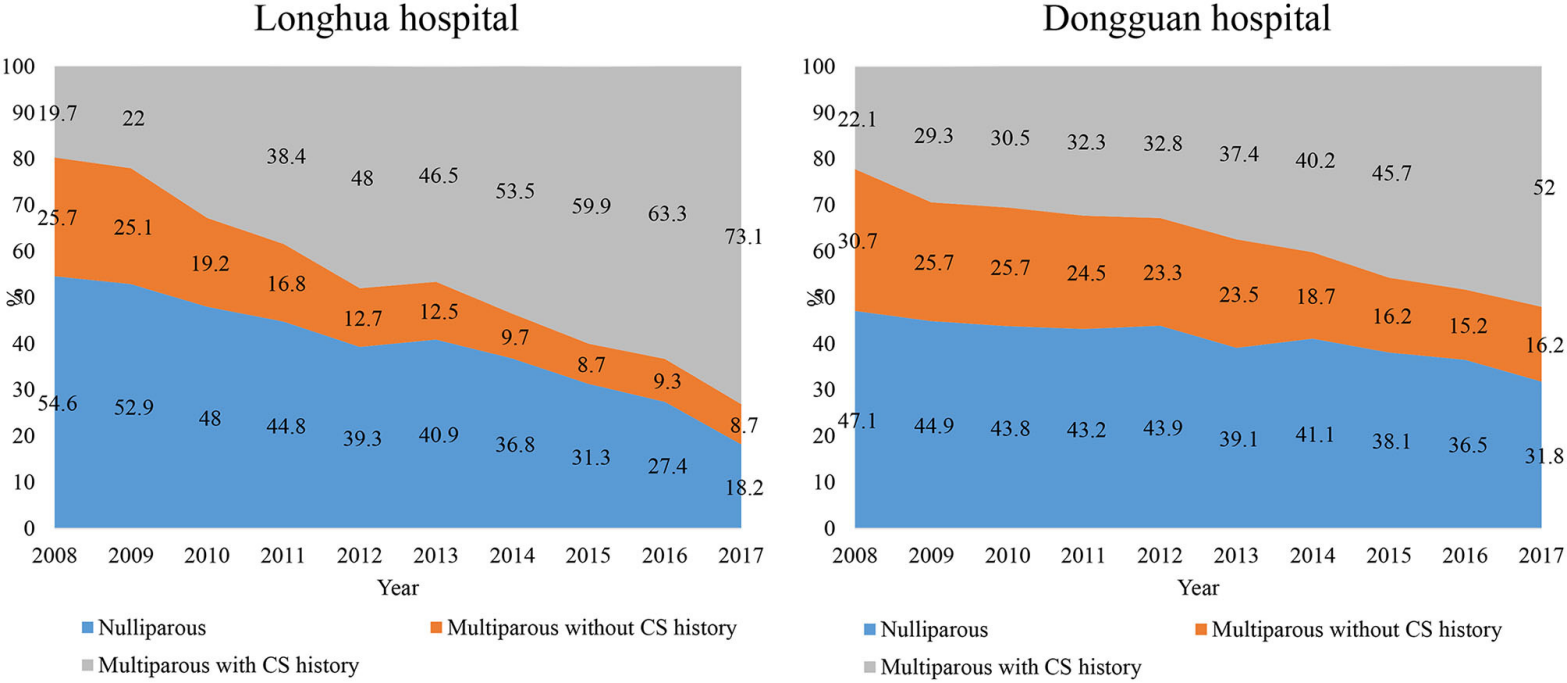
Trends in composition of CS by parity and history of CS in 10 years from 2008 to 2017 in two hospitals.

Figure 4 showed the trends in composition of CS cases by parity and history of CS. In 2008, the composition of CS cases was comparable between the two study hospitals. For example, 55% in Longhua and 47% in Dongguan of CS cases were nulliparous women. Ten years later, the proportion of nulliparous women accounted for only 18% in Longhua but 32% in Dongguan. Analyses in Robson subgroups showed that the proportion for Robson class 1 and 2 groups decreased significantly, while that for other Robson subgroups all increased in Longhua. In Dongguan, the proportion for Robson subgroups showed a similar pattern as that in Longhua except that for Robson class 4–7 group, which remained unchanged (Supplementary Tables 1, 2).

### Reasons for No Trial of Vaginal Trial and Trial Failure

About 20% of women did not try vaginal birth in both the hospitals, but women in Dongguan were more likely due to the women's will (20.0 vs. 11.7%), while women in Longhua were more likely due to scarred uterus and severe complications (Table 4). For those women who tried in Dongguan were more likely to fail (6.7 vs. 2.8%). Among the reasons of the failure, women in Dongguan were twice likely to be called off by the women herself or families (32.2 vs. 14.7%), while women in Longhua were more likely due to abnormal stage of labor.

**Table 4:** Vaginal birth trial and reasons for no trial and trial failure in two hospitals in Guangdong, China.

**Characteristics**	**Classification**	**Longhua (*****N*** **=** **42,441)**				**Dongguan (*****N*** **=** **36,935)**				***P*** **-value**		
		**All**	**Without**	**With**		**All**	**Without**	**With**		**All**	**Without**	**With**
			**uterine scar**	**uterine scar**			**uterine scar**	**uterine scar**			**uterine scar**	**uterine scar**
Trial of vaginal birth and actual delivery mode	Yes and succeed	32,942 (77.6)	31,303 (85.2)	1,639 (28.9)		26,728 (72.4)	25,896 (80.0)	832 (18.3)		<0.0001	<0.0001	<0.0001
	Yes but failed, CS	1,168 (2.8)	1,148 (3.1)	20 (0.4)		2,488 (6.7)	2,307 (7.1)	181 (4.0)				
	No, CS	8,331 (19.6)	4,312 (11.7)	4,019 (70.8)		7,719 (20.9)	4,182 (12.9)	3,537 (77.7)				
The reason for no trial of vaginal birth	Personal willingness	976 (11.7)	975 (22.6)	1 (0.0)		1,540 (20.0)	1,540 (36.8)	0 (0.0)		<0.0001	<0.0001	<0.0001
	Contracted pelvis	58 (0.7)	57 (1.3)	1 (0.0)		68 (0.9)	68 (1.6)	0 (0.0)				
	Fetal macrosomia	420 (5.0)	379 (8.8)	41 (1.0)		326 (4.2)	326 (7.8)	0 (0.0)				
	Malposition	962 (11.5)	864 (20.0)	98 (2.4)		1,027 (13.3)	924 (22.1)	103 (2.9)				
	Fetal distress	581 (7.0)	546 (12.7)	35 (0.9)		200 (2.6)	199 (4.8)	1 (0.0)				
	Scarred uterus	3,796 (45.6)	46 (1.1)	3,750 (93.3)		3,358 (43.5)	51 (1.2)	3,307 (93.5)				
	Placenta previa	217 (2.6)	177 (4.1)	40 (1.0)		323 (4.2)	252 (6.0)	71 (2.0)				
	Abruption placentae	130 (1.6)	115 (2.7)	15 (0.4)		111 (1.4)	88 (2.1)	23 (0.7)				
	Cord around neck	143 (1.7)	143 (3.3)	0 (0.0)		38 (0.5)	38 (0.9)	0 (0.0)				
	Severe complications	824 (9.9)	795 (18.4)	29 (0.7)		517 (6.7)	502 (12.0)	15 (0.4)				
	Other	224 (2.7)	215 (5.0)	9 (0.2)		211 (2.7)	194 (4.6)	17 (0.5)				
Reason for failure of vaginal delivery	Request by patients or family members	172 (14.7)	168 (14.6)	4 (20.0)		801 (32.2)	695 (30.1)	106 (58.6)		<0.0001	<0.0001	<0.0001
	Fetal distress	448 (38.3)	445 (38.7)	3 (15.0)		936 (37.6)	904 (39.2)	32 (17.7)				
	Abnormal stage of labor	473 (40.4)	467 (40.6)	6 (30.0)		666 (26.8)	635 (27.5)	31 (17.1)				
	Others	78 (6.7)	71 (6.2)	7 (35.0)		85 (3.4)	73 (3.2)	12 (6.6)				

### Comparison Between the Two Study Hospitals in Complications

The compatible data of complications showed that there were no differences in number of neonatal and maternal deaths and hysterorrhexis between the two study hospitals. The number of women who experienced hysterectomy and transfusion was slightly higher in Dongguan but with no clinical significance (Table 5).

**Table 5:** Comparison between two study hospitals in women and neonatal complications.

**Major**	**Dongguan hospital**	**Longhua hospital**	***P*-value**
**complications**	**(*N* = 36,935)**	**(*N* = 42,441)**	
Neonatal death	8 (0.02)	11 (0.03)	0.70
Maternal death	3 (0.01)	2 (0.01)	0.55
Hysterorrhexis	9 (0.02)	6 (0.01)	0.30
Hysterectomy	13 (0.04)	5 (0.01)	0.03
Transfusion	630 (1.71)	639 (1.51)	0.03

*Data shown in the table are number of cases and % of each complication event*.

## Discussions

With tremendous efforts, China's national CS rate reduced significantly for about 5% from 2014 to 2016 (8, 10, 11). However, the average CS rate in China was still high, at around 40% (10). There were a lot of doubts that if the WHO's ideal goal of CS rate at 10–15% is achievable. It is particularly challenging for China, because its previous high CS rate and the relaxation of one-child policy would jointly lead to a significantly increased proportion of delivery women with a scar uterus.

Our study chose two typical regional hospitals in China. The CS rate standardized to age, education level, parity, and history of CS in Longhua hospital reduced sharply and steadily since 2010, when the first national policy was issued, from about 35% down to 13% in 2016, when the universal two-child policy was formally implemented. Although it leveled off then but remained well in the ideal range of the WHO-recommended goal. While the standardized rate in Dongguan hospital varied around 25% at the beginning, increased to 36% in 2011, decreased sharply to 27% in 2012, leveled off until 2015 (27.6%), and then bounced back to 35% in 2017. During the 10 years of observation, no difference was found in major maternal complications and neonatal deaths between the two study hospitals.

Under the same national policy, the performance of the two study hospitals governed by two different local health authorities varied significantly, in terms of taking actions to reduce CS rate. This variation matched well with the trend of standardized CS rate in the two hospitals. For example, the obstetric department director in Longhua hospital was personally very supportive to vaginal birth, and hence, immediately started to train the team and promote using midwifery techniques when the first national policy was issued. Empowering midwives to manage midwifery techniques by sequential training session is an effective intervention to reduce the CS rate (17, 18). Midwifery techniques were also recommended in “Implementation plan of the Chinese women and children development guidelines in 2011–2020,” which was issued by Ministry of Health (MOH) in 2012. The local health bureau of Shenzhen, which oversights Longhua hospital, not only put the non-medical CS rate as an indicator for hospital performance appraisal but also spot-checked the indication on quarterly basis. To comply with the local policy, the department director announced the rule that any non-medical CS must be approved before implementation. In contrast, the department director of Dongguan hospital responded to the call of the state to reduce CS through group meetings and communications, and took no more other administrative actions. The CS can increase the comorbidity risks for women and newborns when done without medical indication, and non-indicated CS accounted for the majority of cesarean deliveries (19, 20). Avoiding medically unnecessary CS operations is the effective method to control the high CS rate (20). The director was interested in the technical method to reduce CS among women with a scar uterus and the research program initiated since 2012. Since then, the CS rate also reduced in Dongguan, for about 5%. The local health authority also put the non-medical CS rate as an indicator for hospital appraisal but no site monitoring was carried out.

A detailed analysis supported that Longhua implemented the national and local policies more strictly and followed the clinical guidelines more precisely than Dongguan. For example, although women in both the hospitals had similar proportion (20%) of no-try vaginal birth, women in Dongguan were twice likely due to the women's will (20.0 vs. 11.7%), while women in Longhua were more likely due to scarred uterus and severe complications. For those women who tried in Dongguan were more likely to fail (6.7 vs. 2.8%). Among the reasons of the failure, women in Dongguan were twice likely to be called off by the women herself or families (32 vs. 15%), while women in Longhua were more likely due to abnormal stage of labor.

The women who gave birth in these two hospitals were comparable in terms of the proportion with Robson classification 3 and above and proportion with age over 35. Besides, the proportion of multiparous women with a scar uterus increased rapidly in both the hospitals and at the same pace, from about 7% in 2008 to 20% in 2017. These proportions are consistent with those found through NMNMSS (10). The repeat CS is clearly a new challenge for the reduction of CS rate in China. As shown in our study, the proportion of CS cases attributed to Robson class 3 (single, cephalic, ≥37 weeks of gestation with uterine scar) increased from 19 to 55% over the 10 years. To achieve the reduction in CS rate, vaginal birth after CS (VBAC) has been encouraged in the two hospitals since 2012, and the repeat CS rate dropped by half in Longhua and by about 6% in Dongguan. We estimated that about 6% absolute reduction in overall CS rate in Longhua and about 1% absolute reduction in Longhua was attributed to the dramatic reduction of the repeat CS. In comparison with the two hospitals, the weighted rate of VBAC in China was only 9.6% between 2012 and 2016 according to NMNMSS data (12). That will translate to only 2% reduction in the overall CS rate reduction. The low VBAC rate was believed due to concerning the risk of complications associated with VBAC. Our team previously reported a relatively high success rate of VBAC (84%) along with a low incidence of serious complications (0.3% uterine rupture) (21). VBAC was proved to be an accepted practice and contributed to reducing repeat CS in many countries (22, 23). We believe the expert consensus on implementing VBAC issued by the Chinese Society of Obstetrics and Gynecology in 2016 would help to promote VBAC to a larger scale in China (24). It will be interesting to keep looking on the trends in VBAC use at the country level after the expert consensus issued (25).

Besides the efforts on the reduction of repeat CS, Longhua also made a lot to reduce CS among nulliparous women as well as multiparous women without the history of CS. We found various reasons for these high CS rate in China, and for the variations among the hospitals, and sometimes even in the same cities. In different hospitals, one can find different populations, which explain the differences; these cannot be explained by the modified Robson classification. The reasons underling CS decision-making are complex, involving pregnant women, their families, health-care providers (fear of the obstetricians from possible lawsuits), and contextual factors. A systematic review reported that women preferred CS due to perceived consequences of vaginal delivery or fear of pain during labor (26, 27). In our study, personal willingness was the leading cause for CS except for scarred uterus, and more than one quarter of CS were required by patients or family members after trying vaginal birth. Health education that aimed to increase knowledge on the advantages of vaginal birth should be strengthened through an intensive publicity campaign. The proportion of women with over 9 years of education was significantly higher in Longhua; this may also explain why CS rate reduction in all subgroups in Longhua was more apparent, comparing with its counterpart.

The impact of full relaxation of the one-child policy in China was generally negative and increased the CS rate in all women in Dongguan after 2015. In Longhua, the CS rate also increased in multiparous women with a scar uterus, leveled off in multiparous women without a scar uterus, and kept decreasing in nulliparous women. Again, it should represent the differences in the two hospitals in implementing the national policy on CS reduction, but it might also reflect the increase of women with older age for the second delivery due to the relaxation of one-child policy. A recent report of national database also indicated the same trend after universal two-child policy was issued in 2015 (10).

Our study has several limitations. First, the study included only two hospitals and should not represent all hospitals in China. In particular, both Shenzhen and Dongguan are economically developed cities, and many of the study participants in these two hospitals were immigrants. However, we believe that the successful experiences from Longhua should be applicable to other hospitals in mainland China, if the actions and local policies that were taken by Longhua hospital and the Shenzhen health authority for strict implementation of the national policies and clinical guidelines/consensus could be replicated. Considering the geography and population size, it may still require a long time to achieve the WHO's goal nationwide in China. How the policies are implemented elsewhere in China is unknown. Whether the Longhua experiences are generalizable to other hospitals in other part of the world remain to be proved, though it does not hurt the comparability between the participants from the two hospitals. Second, since we were unable to separate women who had spontaneous labor from those who had CS before labor or delivered after induction, we used the modified Robson classification for our analysis. Because these groups account only small proportion of all delivery women, it should not affect our study results significantly. Finally, the participants came from different hospitals and there were significant differences in certain demographic and clinical characteristics, leading to the inevitable confounding bias. To control these important potential confounding biases, we compared the trend of CS rate over 10 years in the two hospitals by standardizing the CS rate with age, education level, parity, CS history, and Robson classification.

## Conclusions

Our study demonstrated that the ideal goal recommended by the WHO for CS reduction is achievable in China, without increase in major maternal complications and neonatal deaths. The execution ability of local governments and hospitals, and the obstetric department director in particular in implementing the national policies and clinical guidelines played a critical role for the success. These important and valuable national policies should be persistently promoted and supervised around the country, including midwifery techniques training and non-medical CS approval (obligatory documented second opinion before any operation except for obvious emergencies).

## Data Availability Statement

The raw data supporting the conclusions of this article will be made available by the authors, without undue reservation.

## Ethics Statement

The studies involving human participants were reviewed and approved by the Institutional Review Boards of The First Dongguan Affiliated Hospital of Guangdong Medical University. Written informed consent for participation was not required for this study in accordance with the national legislation and the institutional requirements.

## Author Contributions

Y-JJ, H-BW, Y-XL, Y-FW, and HY contributed to the development of the study protocol and took responsibility for the accuracy of the data analysis. Y-JJ, H-BW, and Y-XL were the principal investigators and managed the protocol. Y-JJ, H-BW, ZB, D-JL, KM, JY, Y-XL, Y-FW, and HY were involved in the initial draft of the manuscript. H-BW was responsible for data management and statistical analysis. All authors had full access to all of the data in the study, critically revised the manuscript for important intellectual content, and gave approval for final manuscript.

## Funding

The study was funded by Guangdong Provincial Science and Technology Plan (2017A020214007) and Dongguan Technology Development Society (201950715032188).

## Conflict of Interest

The authors declare that the research was conducted in the absence of any commercial or financial relationships that could be construed as a potential conflict of interest.

## Publisher's Note

All claims expressed in this article are solely those of the authors and do not necessarily represent those of their affiliated organizations, or those of the publisher, the editors and the reviewers. Any product that may be evaluated in this article, or claim that may be made by its manufacturer, is not guaranteed or endorsed by the publisher.

## Abbreviations

CS: cesarean sections
WHO: World Health Organization
VBAC: vaginal birth after CS
cOR: crude odds ratio
CMCHA: China Maternal and Child Health Association
MOH: Ministry of Health
NHFPC: National Health and Family Planning Commission
CSOG: Chinese Society of Obstetrics and Gynecology
SC: State Council
NMNMSS: National Maternal Near Miss Surveillance System.
