# Supplementary material for: Achieving WHO's Goal for Reducing Cesarean Section Rate in a Chinese Hospital
Source: Front Med (Lausanne). 2021 Nov 22;8:774487. doi: 10.3389/fmed.2021.774487 (PMC8645788; doi:10.3389/fmed.2021.774487)
Supplement: Supplementary file 1 [file Table_1.DOCX]

Supplement Table 1 Cesarean section rate (R%) in each year in each Robson group and proportion of total CS number attributed to each group (P%), Longhua hospital

| Year | All subjects  (N=42441) | | Robson’s class 1 (N=18027) | | | Robson’s class 2 (N=15755) | | | Robson’s class 3 (N=5166) | | | Robson’s class 4-7  (N=1505) | | | Robson’s class 8  (N=1988) | | |
| --- | --- | --- | --- | --- | --- | --- | --- | --- | --- | --- | --- | --- | --- | --- | --- | --- | --- |
|  | n | R% | n | R% | P% | n | R% | P% | n | R% | P% | n | R% | P% | n | R% | P% |
| 2008 | 1260 | 37.9 | 616 | 38.2 | 48.9 | 266 | 22.0 | 21.1 | 222 | 98.2 | 17.6 | 110 | 92.4 | 8.7 | 46 | 28.6 | 3.7 |
| 2009 | 1182 | 37.3 | 546 | 37.1 | 46.2 | 236 | 20.0 | 20.0 | 233 | 99.1 | 19.7 | 141 | 91.6 | 11.9 | 26 | 20.5 | 2.2 |
| 2010 | 1044 | 28.1 | 430 | 24.7 | 41.2 | 137 | 10.3 | 13.1 | 312 | 98.4 | 29.9 | 119 | 89.5 | 11.4 | 46 | 23.6 | 4.4 |
| 2011 | 1041 | 25.6 | 386 | 20.1 | 37.1 | 116 | 8.2 | 11.1 | 371 | 95.4 | 35.6 | 141 | 81.0 | 13.5 | 27 | 15.0 | 2.6 |
| 2012 | 1040 | 21.4 | 304 | 13.9 | 29.2 | 79 | 4.6 | 7.6 | 463 | 83.6 | 44.5 | 162 | 86.6 | 15.6 | 32 | 13.5 | 3.1 |
| 2013 | 950 | 21.8 | 292 | 15.1 | 30.7 | 81 | 5.2 | 8.5 | 393 | 79.6 | 41.4 | 138 | 89.0 | 14.5 | 46 | 21.4 | 4.8 |
| 2014 | 936 | 20.1 | 230 | 11.6 | 24.6 | 49 | 3.0 | 5.2 | 451 | 69.8 | 48.2 | 166 | 87.4 | 17.7 | 40 | 18.2 | 4.3 |
| 2015 | 689 | 16.2 | 136 | 8.0 | 19.7 | 29 | 1.8 | 4.2 | 373 | 62.0 | 54.1 | 107 | 84.9 | 15.5 | 44 | 21.8 | 6.4 |
| 2016 | 599 | 12.4 | 113 | 6.3 | 18.9 | 23 | 1.2 | 3.8 | 330 | 44.9 | 55.1 | 96 | 79.3 | 16.0 | 37 | 15.4 | 6.2 |
| 2017 | 758 | 14.6 | 89 | 5.2 | 11.7 | 25 | 1.1 | 3.3 | 500 | 51.7 | 66.0 | 112 | 76.7 | 14.8 | 32 | 15.2 | 4.2 |
| P_trend_ |  |  |  |  |  |  |  |  |  |  |  |  |  |  |  |  |  |
| All | 9499 | 22.4 | 3142 | 17.4 | 33.1 | 1041 | 6.6 | 11.0 | 3648 | 70.6 | 38.4 | 1292 | 85.9 | 13.6 | 376 | 18.9 | 4.0 |

R%: Number of CS divided by total number of women in the group; P%: Number of CS in the group divided by total number of CS in each year.

Robson’ class 1: Nulliparous, single, cephalic, ≥37 weeks; Robson’s class 2: Multiparous, single, cephalic, ≥37 weeks, without uterine scar;

Robson’s class 3: Uterine scar, single, cephalic, ≥37 weeks; Robson’s class 4-7 (Nulliparous, single, breech/multiparous, single, breech/all multiple pregnancies/all single other abnormal lies; Robson’s class 8: All single, cephalic, ≤36 weeks.

Supplement Table 2. Cesarean section rate (R%) in each year in each Robson group and proportion of total CS number attributed to each group (P%), Dongguan Hospital

| Year | All subjects  (N=36935) | | Robson’ class 1 (N=13790) | | | Robson’s class 2 (N=15876) | | | Robson’s class 3 (N=4130) | | | Robson’s class 4-7  (N=1647) | | | Robson’s class 8 (N=1492) | | |
| --- | --- | --- | --- | --- | --- | --- | --- | --- | --- | --- | --- | --- | --- | --- | --- | --- | --- |
|  | n | R% | n | R% | P% | n | R% | P% | n | R% | P% | n | R% | P% | n | R% | P% |
| 2008 | 1156 | 25.8 | 465 | 24.9 | 40.2 | 252 | 13.1 | 21.8 | 231 | 91.7 | 20.0 | 171 | 79.2 | 14.8 | 37 | 17.5 | 3.2 |
| 2009 | 979 | 25.0 | 365 | 22.3 | 37.3 | 202 | 11.8 | 20.6 | 261 | 92.9 | 26.7 | 129 | 82.7 | 13.2 | 22 | 17.3 | 2.2 |
| 2010 | 1100 | 26.2 | 395 | 23.4 | 35.9 | 214 | 11.5 | 19.5 | 312 | 95.4 | 28.4 | 149 | 80.5 | 13.5 | 30 | 20.3 | 2.7 |
| 2011 | 1206 | 28.7 | 431 | 26.9 | 35.7 | 219 | 11.7 | 18.2 | 359 | 94.2 | 29.8 | 143 | 86.1 | 11.9 | 54 | 31.6 | 4.5 |
| 2012 | 1164 | 26.2 | 390 | 23.6 | 33.5 | 173 | 9.1 | 14.9 | 343 | 70.4 | 29.5 | 200 | 93.5 | 17.2 | 58 | 33.0 | 5.0 |
| 2013 | 924 | 26.2 | 290 | 22.5 | 31.4 | 139 | 9.2 | 15.0 | 307 | 74.3 | 33.2 | 145 | 89.0 | 15.7 | 43 | 29.5 | 4.7 |
| 2014 | 908 | 27.7 | 299 | 25.8 | 32.9 | 116 | 8.3 | 12.8 | 321 | 76.8 | 35.4 | 127 | 90.7 | 14.0 | 45 | 28.0 | 5.0 |
| 2015 | 779 | 27.2 | 232 | 23.7 | 29.8 | 77 | 6.3 | 9.9 | 316 | 71.2 | 40.6 | 113 | 92.6 | 14.5 | 41 | 39.0 | 5.3 |
| 2016 | 924 | 30.7 | 258 | 26.0 | 27.9 | 77 | 6.4 | 8.3 | 402 | 75.6 | 43.5 | 140 | 94.6 | 15.2 | 47 | 38.2 | 5.1 |
| 2017 | 1067 | 35.3 | 278 | 30.3 | 26.1 | 109 | 8.7 | 10.2 | 500 | 84.0 | 46.9 | 129 | 94.2 | 12.1 | 51 | 41.1 | 4.8 |
| P_trend_ |  |  |  |  |  |  |  |  |  |  |  |  |  |  |  |  |  |
| All | 10207 | 27.6 | 3403 | 24.7 | 33.3 | 1578 | 9.9 | 15.5 | 3352 | 81.2 | 32.8 | 1446 | 87.8 | 14.2 | 428 | 28.7 | 4.2 |

R%: Number of CS divided by total number of women in the group; P%: Number of CS in the group divided by total number of CS in each year.

Robson’ class 1: Nulliparous, single, cephalic, ≥37 weeks; Robson’s class 2: Multiparous, single, cephalic, ≥37 weeks, without uterine scar;

Robson’s class 3: Uterine scar, single, cephalic, ≥37 weeks; Robson’s class 4-7 (Nulliparous, single, breech/multiparous, single, breech/all multiple pregnancies/all single other abnormal lies; Robson’s class 8: All single, cephalic, ≤36 weeks.
